# *Salmonella enterica* serovar Typhimurium isolated from the urine of a dog undergoing treatment for immune-mediated polyarthritis

**DOI:** 10.1099/jmmcr.0.005153

**Published:** 2018-05-08

**Authors:** Stephen D. Cole, Shannon M. Palermo, Shelley C. Rankin

**Affiliations:** ^1^​ Department of Pathobiology, School of Veterinary Medicine, University of Pennsylvania, Philadelphia, PA, USA; ^2^​ Department of Clinical Studies - Philadelphia, School of Veterinary Medicine, University of Pennsylvania, Philadelphia, PA, USA

**Keywords:** *Salmonella*, bacteriuria, immune-mediated polyarthritis, diarrhoea

## Abstract

**Introduction:**

In people, *Salmonella* is a common agent of gastroenteritis, but it can also cause extraintestinal disease such as urinary tract infections. In addition, *Salmonella* is often linked to the post-infection development of reactive arthritis. In canines, cases that document extraintestinal *Salmonella* infections or diseases similar to reactive arthritis have not been thoroughly described.

**Case presentation:**

A case of a 5-year-old German shepherd dog with *Salmonella* bacteriuria during treatment for immune-mediated polyarthritis (IMPA) is described. The patient first suffered from a 3 month period of diarrhoea and presented for evaluation of a 2 month history of shifting-leg lameness. A diagnosis of IMPA was made based on cytological examination and negative synovial fluid culture. Treatment with immunosuppressive doses of prednisone lead to clinical resolution of lameness, but on a recheck abnormal urine was noted. *Salmonella enterica* serovar Typhimurium was isolated using standard culture methods. The patient was treated with enrofloxacin to control the bacteriuria.

**Conclusion:**

This case report is, to the best of our knowledge, the first to describe *Salmonella* bacteriuria in a dog and suggests that *Salmonella* infection may be a potential inciting factor for IMPA.

## Introduction

*Salmonella enterica* is a Gram-negative pathogen and a major cause of food-borne illness [1]. Non-typhoidal *Salmonella* serovars typically cause self-limiting diarrhoea in veterinary and human patients, but chronic infections have also been reported [2–4]. Extraintestinal infections are rare in both people and dogs, but may include endocarditis [5], pneumonia [6], discospondylitis [7] and urinary tract infection [8]. In addition, one of many possible sequelae of *Salmonella* enteritis in people is reactive arthritis that is characterized as post-infectious, suppurative inflammation that may affect one or multiple joints [9]. A systematic review calculated a weighted mean incidence of reactive arthritis cases of 12/1000 in human enteric salmonellosis patients [10].

A similar condition called immune-mediated polyarthritis (IMPA) occurs in dogs, but an underlying cause is rarely identified [11]. IMPA is considered a type III hypersensitivity reaction with immune complex deposition [11]. IMPA is divided into four types: idiopathic (I), reactive (II), gastrointestinal (III) and neoplastic (IV) [11]. Specifically, type II has been linked to extra-articular infections (i.e. tick-borne disease, prostatitis, necrotizing pharyngitis) and type III to gastrointestinal or hepatic disease [11]. In the veterinary literature, to the authors' knowledge, there are no case reports of *Salmonella* as an agent of bacteriuria in a dog and no case reports that have linked IMPA with *Salmonella* infection.

## Case report

A 5-year-old, castrated-male German shepherd dog was presented for an approximately 2 month history of alternating hindlimb and forelimb lameness. On physical examination, the carpi and elbows were warm, painful and effusive bilaterally. Cytological examination of synovial fluid obtained via arthrocentesis from the left and right elbows and carpi revealed suppurative inflammation, but no infectious agents were identified. Culture of synovial fluid was negative by aerobic culture. The dog also had intermittent, chronic diarrhoea for approximately 3 months and had lost approximately 23 % (10 kg) of its overall body weight. Faecal culture was negative for *Campylobacter* and *Salmonella*, and a faecal float was free of parasites. The patient was antibody negative for *Ehrlichia*, *Anaplasma* and *Borrelia* by SNAP 4Dx (Idexx Laboratories) and *Bartonella* by Western blot (National Veterinary Laboratory, Franklin Lakes, NJ, USA). Urinalysis was not performed initially. The patient was diagnosed with IMPA and treated with immunosuppressive doses of prednisone (1.1 mg kg^−1^ twice daily for approximately 5 months, including a tapered regimen). Tylosin powder [Elanco (Tylan Powder)] (1/2 teaspoon once daily for 21 days) and omeprazole [Procter and Gamble (Prilosec OTC)] (1 mg kg^−1^ once daily pro re nata) were prescribed for the chronic diarrhoea. The owner also began a commercial, limited ingredient diet (Blue Buffalo).

At recheck, 10 weeks after diagnosis of IMPA, the patient's orthopaedic pain was well managed, the effusion had grossly resolved and the diarrhoea had subsided. While the patient did not exhibit any signs of lower urinary tract disease, malodorous urine was noted during the examination.

## Investigations

A urinalysis revealed dilute urine with pyuria (6–10 per high powered field) and bacteriuria. Quantitative aerobic culture of urine obtained via cystocentesis yielded greater than 100 000 c.f.u. ml^−1^ of a non-lactose-fermenting organism on MacConkey agar (Remel). The organism was biochemically identified as *Salmonella* spp. with the MicroScan Walkway 40si NC31 panel (Dade-Behring, Deerfield, IL). and was sensitive to all antimicrobials tested, including amoxicillin, trimethoprim/sulfamethoxazole and enrofloxacin [12]. The organism was serotyped as *S. enterica* serovar Typhimurium (4,12 : i : 1,2) using the xMAP Salmonella serotyping assay (Luminex).

## Treatment

The patient completed a 21 day course of oral enrofloxacin (Luminex) (10 mg kg^−1^ SID). Although the animal may have had subclinical bacteriuria according the International Society for Companion Animal Infectious Disease guidelines, and therapy may not have been warranted, the clinicians felt it prudent to treat given the zoonotic potential of the pathogen and the immunocompromised nature of the patient [13]. A fluoroquinolone antimicrobial was selected based on previous reports in the human literature [14, 15]. The authors acknowledge that amoxicillin or trimethoprim-sulfamethoxazole may have been a more judicious choice and should be considered in future cases. At the time of treatment, appropriate at-home interventions were put in place to prevent zoonotic transfer to the owners. Specifically, gloves were recommended for handling faecal material, it was recommended that contact with urine be avoided and it was advised that immunocompromised individuals should not be allowed to come into contact with the dog.

## Outcome and follow-up

Seven-day follow-up urine and faecal cultures were negative for *Salmonella*. At the time of writing (approximately 10 months from initial presentation) the patient was clinically normal.

## Discussion

It has been postulated that domestic animals can serve as a reservoir of enteric agents of zoonotic disease (i.e. *Salmonella*, *Campylobacter*, possibly *Clostridium difficile).* A recent multi-laboratory study reported a 3.8 % prevalence of *Salmonella* in the faeces of diarrhoeic dogs, which was significantly different from the 1.8 % in non-diarrhoeic dogs [16]. *S*. *enterica* serovar Typhimurium was not among the serovars isolated [17]. A major risk factor for *Salmonella* infection in dogs is known to be the feeding of raw diets [17, 18]. The patient in this study was fed a common, commercial dry-food diet and there was no history of eating raw food. A single case of *Salmonella* bacteriuria in a cat has been linked to feeding a raw diet [19]. *S. enterica* serovar Typhimurium has not been reported to be one of the most common serotypes from dogs [17, 20], but it has been linked to several outbreaks that involved people and domestic animals (dogs and cats). These outbreaks were linked to cats that hunt wild birds [21], veterinary patients undergoing dental procedures at the same hospital [22] and animal shelters [23].

Definitive diagnosis of the aetiology of the gastrointestinal disease was never made; therefore, classifying the IMPA type is difficult. This patient could traditionally be classified as having type III (gastrointestinal) IMPA. The corticosteroids administered for IMPA may have also treated concurrent inflammatory bowel disease, leading to the resolution of diarrhoea [24]. The authors also suggest that there may be crossover with type II (reactive), since it is possible that *Salmonella* was responsible for the gastrointestinal disease. Although the faecal culture was negative for *Salmonella,* recovery of *Salmonella* from faeces can be variable depending on the technique used [25] and due to intermittent shedding [26]. To rule out *Salmonella* as an agent of enteritis, the American College of Veterinary Internal Medicine consensus statement recommends multiple faecal cultures if molecular detection (PCR) is not performed [16]. In retrospect, an approach such as this may have been helpful to confirm *Salmonella* as the cause of enteritis in this dog. Further research could evaluate the prevalence of *Salmonella* and other enteric pathogens in dogs with IMPA. Infectious agents that may be associated with IMPA in dogs are often vector-borne agents (i.e. *Ehrlichia, Borrelia, Bartonella*) [27], but greater understanding of all pathogens associated with IMPA may help elucidate its pathogenesis. It is likely that corticosteroid-mediated immunosuppression predisposed this patient to ascending bacteriuria [28].

In summary, this case report identifies and describes *Salmonella* from the urinary tract of a dog with chronic diarrhoea and IMPA. Given this report and the relationship of *Salmonella* with reactive arthritis in people, owners should be asked about historical diarrhoea when patients have suspected or diagnosed IMPA. In patients with current or previous diarrhoea, the authors suggest thorough diagnostics such as serial faecal cultures or PCR. Identification of *Salmonella* in patients with IMPA could have important therapeutic implications and allow for implementation of appropriate measures to prevent zoonotic transfer. This report also suggests that the phenomenon of reactive arthritis following *Salmonella* infection may not be limited to people.
